# Risk factors for pneumonitis in patients treated with anti‐programmed death‐1 therapy: A case‐control study

**DOI:** 10.1002/cam4.1579

**Published:** 2018-05-23

**Authors:** Pengfei Cui, Zhefeng Liu, Guoqiang Wang, Junxun Ma, Yuanyu Qian, Fan Zhang, Chun Han, Yaping Long, Ye Li, Xuan Zheng, Danyang Sun, Jing Zhang, Shangli Cai, Shunchang Jiao, Yi Hu

**Affiliations:** ^1^ First Department of Medical Oncology Chinese PLA General Hospital Beijing China; ^2^ Department of Graduate Administration Chinese PLA General Hospital Beijing China; ^3^ The Medical Department 3D Medicines Inc. Shanghai China

**Keywords:** anti‐PD‐1, case‐control study, pneumonitis, risk factors

## Abstract

Immune checkpoint blockade‐related pneumonitis is a rare but potentially life‐threatening adverse effect, but its risk factors are not completely understood. This case‐control study was conducted to identify pneumonitis risk factors in patients treated with anti‐PD1 monoclonal antibodies (mAbs), including all the patients who developed pneumonitis after anti‐PD‐1 mAbs treatment in the Cancer Center of the Chinese People's Liberation Army from September 2015 to September 2017. Two controls per case were matched according to a propensity‐score matching algorithm to account for confounding effects caused by individual baseline variables. Demographic and clinical information was obtained from medical records. In total, 55 cases and 110 controls were included in the study. No association was observed between smoking status or primary lung cancer and risk of pneumonitis. Significant risk factors for pneumonitis related to anti‐PD‐1 mAbs were prior thoracic radiotherapy, prior lung disease and combination therapy with odds ratios of 3.34 (1.51‐7.39), 2.86 (1.45‐5.64) and 2.73 (1.40‐5.31), respectively. The associations remained significant in the multivariable logistic regression model. The risk of pneumonitis induced by anti‐PD‐1 mAbs is associated with prior thoracic radiotherapy, prior lung disease, and combination therapy. Clinicians should monitor these features in patients receiving anti‐PD‐1 therapy to optimize clinical safety and efficacy.

## 1 INTRODUCTION

Recently, substantial progress has been achieved in cancer treatments by directing cytotoxic T‐lymphocyte antigen‐4 (CTLA‐4), programmed death receptor‐1 (PD‐1) or its associated ligand (PD‐L1) to enhance immunologic responses and anti‐tumor activity, which has significantly improved cancer patient prognosis.^1^ Despite the impressive clinical benefits, however, the adverse events concomitant with immune checkpoint blockades (ICBs) cannot be ignored.

Immune checkpoints contribute to the maintenance of immunologic homeostasis. Checkpoint blockade disrupts normal immune homeostasis and results in immune‐related adverse events (irAEs), including dermatologic, hepatic, endocrine, gastrointestinal, and pulmonary complications as well as other less common side effects.^2^, ^3^ Among these irAEs, pneumonitis, which is defined as focal or diffuse inflammation of the lung parenchyma,^4^ is rare but potentially life threatening. In general, the incidence of pneumonitis ranges from 0% to 10%.^5^ Although most events are low grade and are improved/resolved with drug holding/immunosuppression [22], pneumonitis is life threatening in rare cases. In an early‐phase study of an anti‐PD‐1 monoclonal antibody (mAb), pneumonitis accounted for 3 deaths.^6^ Recent studies have discussed the clinical features, diagnosis, and management of pneumonitis related to ICBs thoroughly; however, only a few studies have attempted to identify potential risk factors.

In a case report, 3 of 25 patients who received thoracic radiotherapy and anti‐PD‐1 therapy developed pneumonitis, suggesting a possible association between pneumonitis and prior thoracic radiotherapy.^7^ In several meta‐analyses of published randomized clinical trials, pneumonitis incidence is relatively increased in smokers; non‐small‐cell lung cancer (NSCLC) patients; patients with previous lung disease, including chronic obstructive pulmonary disease; and those who received combination therapy.^8^, ^9^, ^10^ However, these data were not directly compared but were instead extracted from separate randomized controlled studies, and the variability of these randomized clinical trial characteristics, such as inclusion or exclusion criteria, dosage and the stage of disease, may have confounded the results. Radiomic features can classify and predict patients at baseline who will subsequently develop immunotherapy‐induced pneumonitis.^11^ However, small sample sizes in case‐reports and potential confounders in meta‐analyses may limit the reliability and validity of data. Using a larger sample size in the present case‐control study, we aimed to identify the association between factors weakening pulmonary function and the risk of pneumonitis induced by anti‐PD‐1 mAbs.

## 2 MATERIALS AND METHODS

### 2.1 Study population

Eligible patients included those with advanced tumor who developed pneumonitis after anti‐PD‐1 mAbs treatment in the Cancer Center of the Chinese People's Liberation Army from September 2015 to September 2017. Pneumonitis was diagnosed by a radiologist according to computed tomography scans. Patients were excluded if there was a clear alternative etiology, such as proven malignant lung infiltration or active lung infection. Pneumonitis grading was performed by the treating investigators in real time using Common Toxicity Criteria for Adverse Events (version 4.0). Controls were defined as advanced tumor patients treated with anti‐PD‐1 mAbs who did not develop pneumonitis. This study was approved by the Ethics Committee of Chinese People's Liberation Army General Hospital.

### 2.2 Data collection

For all the patients, the following pretreatment demographic and clinical information was obtained from medical records: age, sex, smoking status, weight, metastatic sites, baseline lactic dehydrogenase, previous anticancer treatments, treatment agents with anti‐PD‐1 mAbs, the number of anti‐PD‐1 mAbs treatment cycles, primary tumor type, and prior lung disease. Smoking status was classified as no history of smoking or a positive history. Combination therapy was defined as anti‐PD‐1 treatment with chemotherapy, targeted therapy or CTLA‐4 blockade. Previous anticancer treatment (6 months prior) was classified as chest surgery, thoracic radiotherapy, targeted therapy or chemotherapy. Prior lung disease included pneumothorax, pleural effusion, and pneumonitis before anti‐PD‐1 mAbs therapy. In patients with pneumonitis, the clinical features of pneumonitis were collected retrospectively from medical records. The severity of the pneumonitis was described using the criteria for interstitial lung diseases.^12^, ^13^, ^14^ The median follow‐up time for the case group was 239 days with a range from 12 to 610 days. For the control group, the follow‐up time was 142.5 days with a range from 34 to 793 days.

### 2.3 Statistical analysis

Statistical analyses were performed using SPSS statistical software (version 24.0; SPSS, IBM Corporation, USA). Continuous variables were described with the median (range) and were compared by 2‐sided *t* tests or the Mann‐Whitney *U* test. Categorical variables were compared by Chi‐square test or Fisher's exact test. Propensity‐score matching was used to create case and control groups with similar baseline characteristics. Matching with a ratio of 1:2 was performed using the optimal‐matching algorithm in MatchIt R package 3.4.2.^15^ Propensity scores were estimated by age, sex, weight, metastasis, number of treatment cycles, and baseline lactic dehydrogenase. Estimates of odds ratios (ORs) and 95% confidence intervals (CIs) of risk factors were obtained using logistic regression. Variables with significance were included in a multiple logistic regression analysis. *P *=* *.05 was set as the level of significance. All the reported *P*‐values were 2‐sided.

## 3 RESULTS

### 3.1 Patient characteristics

Overall, 55 cases and 110 controls were included in this analysis. The characteristics of cases and controls are described in Table 1. No differences were observed in age, sex, weight, metastasis, number of treatment cycles and baseline lactic dehydrogenase between the case and control groups after matching.

**Table 1:** Patient demographic and clinical characteristics

Characteristic	Cases (N* *=* *55)	Controls (N* *=* *110)	*P*
Median age, years (range)	53 (26‐77)	57 (23‐87)	.222
Sex, N (%)			
Male	47 (85.5)	84 (76.4)	.174
Female	8 (14.5)	26 (23.6)	
Median weight, kg (range)	70.0 (44.3‐100.0)	69.0 (42.0‐98.0)	.240
Metastasis, N (%)			.457
Yes	51 (92.7)	98 (89.1)	
No	4 (7.3)	12 (10.9)	
No. of treatment cycles of anti‐PD‐1 mAbs, mean (95% CI)	4.0 (3.3‐4.7)	4.4 (4.0‐4.9)	.110
Median baseline LDH, U/L(range)	209 (106.4‐870.4)	188.8 (102.9‐2001.0)	.648
Anti‐PD‐1 mAbs, N (%)			.485
Pembrolizumab	16 (29.1)	39 (35.5)	
Nivolumab	39 (70.9)	71 (64.5)	
Combined therapy			
Targeted therapy	15 (27.3%)	19 (17.3%)	
Chemotherapy	27 (49.1%)	35 (31.8%)	
Anti‐CTLA‐4 therapy	4 (7.3%)	3 (2.7%)	
Cancer type, N (%)			
Adrenal carcinoma	1 (1.8%)	0 (0%)	
Breast cancer	1 (1.8%)	0 (0%)	
Carcinoma of ampulla	0 (0%)	3 (2.7%)	
Carcinoma of fallopian tube	0 (0%)	1 (0.9%)	
Carcinoma of gallbladder	0 (0%)	3 (2.7%)	
Carcinoma of vulva	0 (0%)	1 (0.9%)	
Cervical carcinoma	0 (0%)	1 (0.9%)	
Cholangiocarcinoma	0 (0%)	4 (3.6%)	
Colorectal cancer	2 (3.6%)	4 (3.6%)	
Duodenal carcinoma	1 (1.8%)	0 (0%)	
Esophageal carcinoma	3 (5.5%)	4 (3.6%)	
Gastric carcinoma	4 (7.3%)	7 (6.4%)	
Laryngocarcinoma	1 (1.8%)	1 (0.9%)	
Liver cancer	1 (1.8%)	5 (4.5%)	
Lymphoma	4 (7.3%)	7 (6.4%)	
Melanoma	1 (1.8%)	2 (1.8%)	
Non‐small‐cell lung carcinoma	21 (38.2%)	40 (36.4%)	
Ovarian carcinoma	1 (1.8%)	7 (6.4%)	
Pancreatic cancer	6 (10.9%)	6 (5.4%)	
Prostate carcinoma	0 (0%)	2 (1.8%)	
Renal carcinoma	0 (0%)	1 (0.9%)	
Small‐cell lung carcinoma	7 (12.7%)	7 (6.4%)	
Sarcoma	1 (1.8%)	1 (0.9%)	
Urothelial carcinoma	0 (0%)	3 (2.7%)	

LDH, Lactic dehydrogenase.

The clinical features of pneumonitis are summarized in Table 2. In brief, the median time to onset for pneumonitis was 85 days, with a wide range from 2 to 277 days. The quantity (frequency) of patients who developed grade 1‐5 pneumonitis was 30 (54.5%), 17 (30.9%), 6 (10.9%), 1 (1.8%), and 1 (1.8%), respectively. Radiologic severity at the time of pneumonitis was classified as mild (27 of 55 [49.1%]), moderate (22 of 55 [40%]), or severe (6 of 55 [10.9%]). The radiologic subtype was identified as cryptogenic organizing pneumonia‐like pneumonitis (33 of 55 [60%]), ground glass opacities (10 of 55 [18.2%]), interstitial (5 of 55 [9.1%]), hypersensitivity (6 of 55 [10.9%]) and not otherwise specified pneumonitis (1 of 55 [1.8%]).

**Table 2:** Summary of the clinical features of pneumonitis

Clinical features	Cases
Time to pneumonitis onset after the administration of PD‐1 mAbs (days), median (range)	85 (2‐277)
Symptomatic pneumonitis, N (%)	26 (47.3)
Highest treatment required for pneumonitis management, N (%)	
Treatment hold	25 (45.5)
Oral corticosteroids	2 (3.6)
Intravenous corticosteroids	12 (21.8)
Time to management from pneumonitis onset (days), median (range)	1 (0‐13)
Radiologic Subtypes, N (%)	
Cryptogenic organizing pneumonitis‐like	33 (60%)
Ground glass opacities	10 (18.2%)
Interstitial	5 (9.1%)
Hypersensitivity	6 (10.9%)
Pneumonitis not otherwise specified	1 (1.8%)
CTCAE grade	
1	30
2	17
3	6
4	1
5	1
Recurrent pneumonitis, N (%)	3 (5.5)
Radiologic severity, N (%)	
Mild	27 (49.1%)
Moderate	22 (40%)
Severe	6 (10.9%)
Treatment	
No management	2 (3.6%)
Hold treatment	25 (45.5%)
Intravenous corticosteroids	12 (21.8%)
Oral corticosteroids	2 (3.6%)
Unknown	14 (25.5%)

CTCAE, Common toxicity criteria for adverse events.

Treatment was performed on 45.5% (25 of 55) patients, and 25.5% (14 of 55) patients received oral or intravenous steroid therapy. Pneumonitis was improved, resolved, or unchanged in 61.8% (34 of 55) of cases, and it was improved or resolved in 12 of 14 patients (85.7%) who received steroid therapy. Two patients died during the course of pneumonitis treatment.

### 3.2 Risk factors for anti‐PD‐1 mAbs‐related pneumonitis

Immune‐mediated pneumonitis is likely to preferentially attack patients with characteristics that worsen pulmonary conditions, including smoking status, prior treatment, combination therapy, primary tumor type, and prior lung disease. Therefore, we performed logistic regression to identify the association between the occurrence of ICB‐related pneumonitis and these selected variables. However, no prominent association was observed between the risk of pneumonitis and smoking status or lung cancer. Significant factors associated with anti‐PD‐1 mAbs‐related pneumonitis included prior thoracic radiotherapy, prior lung disease and combination therapy (*P *<* *.01). The odds ratios were 3.34 (1.51‐7.39), 2.86 (1.45‐5.64) and 2.73 (1.40‐5.31), respectively (Table 3). Next, prior thoracic radiotherapy, prior lung disease, and combination therapy were included in multiple logistic regression analysis. Similar results were observed as prior thoracic radiotherapy, prior lung disease, and combination therapy were significantly associated with the risk of pneumonitis (*P *<* *.01, Table 4).

**Table 3:** Risk factors for pneumonitis in patients treated with anti‐programmed death‐1 therapy

Characteristics	Cases (N* *=* *55)	Controls (N* *=* *110)	OR		
			OR	95% CI	*P*
Smoking					
Never	23 (41.8)	48 (43.6)			
Ever	32 (58.2)	62 (56.4)	1.08	0.56‐2.07	.824
Prior chemotherapy					
No	10 (18.2)	22 (20.0)			
Yes	45 (81.8)	88 (80.0)	1.13	0.49‐2.58	.781
Prior chest surgery					
No	52 (94.5)	102 (92.7)			
Yes	3 (5.5)	8 (7.3)	0.74	0.19‐2.89	.660
Prior thoracic radiotherapy					
No	37 (67.3)	96 (87.3)			
Yes	18 (32.7)	14 (12.7)	3.34	1.51‐7.39	.003
Prior targeted therapy					
No	34 (61.8)	67 (60.9)			
Yes	21 (38.2)	43 (39.1)	0.96	0.50‐1.87	.910
Combination therapy					
No	21 (38.2)	69 (62.7)			
Yes	34 (61.8)	41 (37.3)	2.73	1.40‐5.31	.003
Prior lung disease					
No	18 (32.7)	64 (58.2)			
Yes	37 (67.3)	46 (41.8)	2.86	1.45‐5.64	.002
Primary tumor type					
Lung cancer	27 (49.1)	47 (42.7)			
Others	28 (50.9)	63 (57.3)	1.29	0.67‐2.48	.439

**Table 4:** Risk factors for pneumonitis identified by multiple logistic regression analysis

Variable	OR	95% CI	*P*
Prior thoracic radiotherapy	3.33	1.39‐7.97	.007
Prior lung disease	2.82	1.36‐5.84	.005
Combination therapy	3.42	1.65‐7.09	.001

## 4 DISCUSSION

In this case‐control study, risk factors, including prior thoracic radiotherapy, prior lung disease, and combination therapy, for pneumonitis induced by anti‐PD‐1 therapy were identified. No significant association was observed between smoking or lung cancer and the risk of anti‐PD‐1 therapy‐related pneumonitis.

As lungs represent the core organ affected by immune‐mediated pneumonitis, factors influencing pulmonary functions may be associated with immune‐mediated pneumonitis. Previous meta‐analyses have demonstrated that NSCLC patients exhibit an increased incidence of pneumonitis compared with that in melanoma patients.^9^, ^10^ However, these data were extracted from separate randomized controlled studies and were not compared directly. Clinical study variables, such as inclusion or exclusion criteria, dosage, and stage, varied in these randomized trials, potentially limiting an accurate interpretation of the results. In addition, a similar incidence among patients with melanoma or NSCLC for monotherapy or combination therapy was revealed in a previous observational study.^16^ Consistent with this finding, no significant association between lung cancer and ICB‐related pneumonitis was detected in this study. In addition, no relation between smoking status and pneumonitis was revealed. However, the possibility of an association between smoking and pneumonitis cannot be fully excluded because detailed information regarding smoking quantity and duration were not available.

However, we observed an association between pneumonitis and prior thoracic radiotherapy, prior lung disease or combination therapy. Thoracic radiotherapy or prior lung disease were associated with poor pulmonary function. NSCLC patients are typically submitted to thoracic radiotherapy and often exhibit other lung complications. The observed increased incidence of pneumonitis in NSCLC patients from previous studies might be attributed to the poor lung function induced by prior thoracic radiotherapy or NSCLC itself rather than the anti‐PD‐1 therapy. Pneumonitis incidence might increase if anti‐PD‐1 mAb was combined with other agents, such as anti‐CTLA‐4 mAb,^9^ also known to carry a risk of pneumonitis.^2^ In this study, we confirmed these observations and further clarified that anti‐PD‐1 treatment combined with chemotherapy, targeted therapy or CTLA‐4 blockade was also associated with pneumonitis risk, indicating the additive effects of multiple agents on lung‐toxic effects.

However, some limitations of this study should be addressed. First, the number of pneumonitis patients might not be sufficient to provide reliable information concerning the significance of each risk factor. Second, the risk of ICB‐related pneumonitis for each combined therapy (chemotherapy, targeted therapy, CTLA‐4 blockade or radiotherapy) might vary due to distinct pharmacological mechanisms. However, the sample size was not adequate to address each combined therapy individually. Third, drug‐induced pneumonitis remains a diagnosis of exclusion and requires the consideration of competing diagnoses. Fourth, we did not include patients treated with anti‐PD‐L1 mAbs who may share similar risk factors for pneumonitis with anti‐PD‐1 mAbs. Fifth, prior lung disease did not include asthma, chronic obstructive pulmonary disease or interstitial lung disease because this information was not provided. Other limitations common to case‐control studies are the risk of observational bias and confounding bias, which was minimized by applying the propensity‐score matching protocol.

In conclusion, we identified prior thoracic radiotherapy, prior lung disease and combination therapy as risk factors for the life‐threatening pneumonitis observed in patients receiving anti‐PD‐1 immunotherapy. Although the detailed role and mechanism underlying these risk factors for the development of pneumonitis requires further investigation, patients with these characteristics require intensive care in cases of anti‐PD‐1 mAbs‐related pneumonitis. To the best of our knowledge, this is the first case‐control study systematically seeking to identify risk factors for anti‐PD‐1 mAbs‐related pneumonitis development in a Chinese population of multiple types of advanced cancer. This study fills an important gap in the literature and supplements the limited number of published reports on anti‐PD‐1 pneumonitis.^5^, ^16^, ^17^, ^18^

## CONFLICT OF INTEREST

The authors have declared no conflict of interests.
